# Comparison of the tumor immune microenvironment phenotypes in different breast cancers after neoadjuvant therapy

**DOI:** 10.1002/cam4.5207

**Published:** 2022-09-08

**Authors:** Mengxue Han, Jinze Li, Si Wu, Chun Wu, Yongqiang Yu, Yueping Liu

**Affiliations:** ^1^ Department of Pathology The Fourth Hospital of Hebei Medical University Shijiazhuang China

**Keywords:** immune microenvironment, neoadjuvant therapy, PD‐L1, triple‐negative breast cancer, tumor‐infiltrating lymphocytes

## Abstract

Neoadjuvant therapy (NAT) treats early‐stage breast cancers, especially triple‐negative breast cancers (TNBCs). NAT improves pathological complete response (pCR) rates for different breast cancer patients. Recently, immune checkpoint inhibitors that target programmed death 1 (PD‐1) or programmed death ligand 1 (PD‐L1) in combination with NAT have shown antitumor activity in patients with early breast cancer. However, the tumor immune microenvironment (TME) in different subtypes of breast cancers, like TNBC, hormone receptor‐positive (HR+), and human epidermal growth factor receptor 2 amplified (HER2+) and its changes by NAT remain to be fully characterized. We analyzed pre‐NAT tumor biopsies from TNBC (n = 27), HR+ (n = 24), and HER2+ (n = 30) breast cancer patients who received NAT, followed by surgery. The different immune makers (PD‐1, PD‐L1, CD3, and CD8) of tumor‐infiltrating lymphocytes (TILs) were identified with immunofluorescence‐based microenvironment analysis. TILs within cancer parenchyma (iTILs) and in cancer stroma (sTILs) were counted separately. We found that PD‐L1+ cells in tumor and stroma were significantly higher in TNBC patients than in others. PD‐L1+ sTILs were significantly higher in pCR than in non‐pCR patients of all the subtypes. The infiltration scores of B‐cell memory, T‐cell CD4+ memory activated, T‐cell follicular helper, and Macrophage M0 and M1 were relatively higher in TNBC patients, indicating immunoreactive TME in TNBC. Analysis of TCGA‐BRCA RNA‐seq indicated that PD‐L1 was highly expressed in TNBC patients compared with HR+ and HER2+ patients. Higher PD‐L1 expression in TNBC patients was associated with significantly longer overall survival (OS). Our results demonstrated that PD‐L1 expression level of iTILs and sTILs is highest in TNBC among breast cancers. TNBC patients had significantly different immunoreactive TME compared with HR+ and HER2+ patients, suggesting potentially favorable outcomes for immunotherapy in these patients. Also, PD‐L1+ could be a powerful predictor of pCR in TNBC patients after NAT.

## Graphical abstract

We analyzed pre‐NAT tumor biopsies from TNBC, HR+, and HER2+ breast cancer patients who received NAT and compared the different immune makers (PD‐1, PD‐L1, CD3, and CD8) of tumor‐infiltrating lymphocytes (TILs). We found PD‐L1+ cells were significantly higher in TNBC patients than others, and PD‐L1+ cells were higher in pathologic complete response (pCR) than in no‐pCR TNBC patients. Our results demonstrated that TNBC patients had significantly different TME, suggesting a potential role for immunotherapy in these patients.

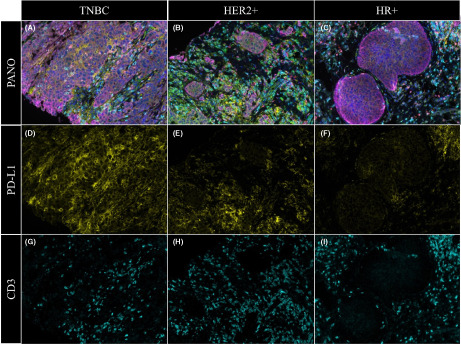

## 1 INTRODUCTION

Female breast cancer (BC) is the most commonly diagnosed cancer worldwide^1^ and the leading cause of cancer deaths in China.^2^ Different molecular subtypes of BC have been recognized, associated with different clinical prognoses and patients' survival.^3^, ^4^ BCs are categorized into three subtypes according to estrogen receptor (ER), progesterone receptor (PR), and human epidermal receptor growth factor 2 (HER2) expression: HR‐positive (ER‐positive and/or PR‐positive), HER2‐positive, and triple‐negative breast cancer (TNBC) which lack expression of ER, PR, and HER2. TNBC has worse overall survival (OS) than non‐TNBC tumors.^5^ Neoadjuvant therapy (NAT), such as neoadjuvant chemotherapy^6^, ^7^, ^8^ and neoadjuvant endocrine therapy,^9^, ^10^ for breast cancer, especially for early breast cancer, is a promising approach and has improved outcomes and changed the standard of care for these subtypes. Strong evidence shows that pathologic complete response (pCR) after neoadjuvant therapy (NAT) is associated with better OS.^11^

Common chemotherapeutic agents used in NAT, such as anthracyclines, taxanes, anti‐HER2 monoclonal antibodies trastuzumab and pertuzumab, can directly induce immunostimulatory effects of tumor cell killing through DC activation.^12^ The capacity for a tumor to initiate a robust immune response appears to be predetermined by baseline immune features.^13^ The tumor‐infiltrating lymphocytes (TILs) and expression of CD8 (cytotoxic T‐cells) and CD4 (T‐helper cells) are valuable prognostic markers in breast cancer.^14^, ^15^, ^16^ PD‐L1 is highly expressed in TNBCs than in non‐TNBC.^17^ However, existing data usually comprise pooled analyses, and the tumor immune microenvironment (TME) of different BC subtypes is largely unknown.^18^

Furthermore, recent trials have shown that a subgroup of breast carcinomas has an enhanced response to immune checkpoint inhibitors, which target programmed death 1 (PD‐1) or programmed death ligand 1 (PD‐L1), combined with NAT or conventional chemotherapy.^19^, ^20^, ^21^ A better understanding of TME between different tumor subtypes is essential for developing innovative therapeutic strategies for immune response. To explore potential similarities and differences in varying BC subtypes and identify novel predictors of response to NAT, we investigated biopsy samples from BC patients who received NAT. We compared the TME of HR‐positive, HER2‐positive, and TNBC patients with our institutional samples and TCGA dataset and investigated the role of PD‐L1 in different subtypes.

## 2 MATERIAL AND METHODS

### 2.1 Patients and tumors

Female patients aged 18 years or older with previously untreated stage II to III breast cancers were retrospectively analyzed at the Fourth Hospital of Hebei Medical University between Jan 2020 and Dec 2020. All patients received NAT and were treated according to national guidelines. Patients underwent a pretreatment core‐needle biopsy prior to initiating NAT. The disease response was monitored with breast imaging (ultrasound and MRI), and tumor response assessment was based on Response Evaluation Criteria in Solid Tumors (RECIST) version 1.1.^22^

Clinical data were obtained from the electronic medical health records. Clinicopathologic data, including the patient's age, tumor size, pathological stage, lymph node metastasis, histologic grade, and Ki‐67 proliferation index, were recorded. For Ki‐67, the percentage of tumor cells with any nuclear staining of any intensity was recorded. Two independent pathologists reviewed all the tumors to confirm the histological diagnosis. Pathological or clinical staging was based on the seventh edition of the American Joint Committee on Cancer.^23^ Pathological subtypes were classified into three groups: HR‐positive breast cancer (HR+, tumors positive for ER or PR); HER2‐positive breast cancer (HER2+, tumors positive for HER2, negative for ER and PR); triple‐negative breast cancer (TNBC, tumors negative for ER, PR, and HER2). HER2+ patients were offered NAT with taxanes in combination with trastuzumab and pertuzumab. TNBC patients received NAT chemotherapy comprised of anthracyclines and taxanes. HR+ patients were offered NAT chemotherapy with anthracyclines and taxanes or taxane in combination with trastuzumab and pertuzumab when HER2 was positive.

Residual cancer burden (RCB)^24^ was retrospectively reviewed by a single pathologist and was used to assess pathologic response to evaluate residual tumor cells. The definition of pCR was noninvasive residual disease in the breast and axillary lymph nodes. The study protocol was approved by the ethics committee of the Fourth Hospital of Hebei Medical University (no. 2022KY05P).

### 2.2 Pathological assessment

The percentage of TILs infiltration was determined on hematoxylin and eosin‐stained slides by breast pathologists according to standardized methods described by the International TILs Working Group.^25^ Tumor tissues from core biopsies pre‐NAT were used to perform immunofluorescence‐based Tissue Microenvironment Analysis Panel (MAP). Multiplex staining for PD‐1, PD‐L1, CD3, CD8, and panCK was performed with PANO 7‐plex IHC kit (cat. 0004100100, Panovue) according to the manufacturer's protocol. In brief, formalin‐fixed, paraffin‐embedded tissue sections were deparaffinized, rehydrated, and subjected to antigen retrieval buffer treatment (Citric acid solution, pH 6.0/pH 9.0). After endogenous peroxidase activity quenched by blocking solution, the sections were then incubated with each primary antibody sequentially, followed by horseradish peroxidase (HRP)‐coupled secondary antibody incubation and tyrosamine signal amplification (TSA).^26^ For each additional marker, the sections were subjected to antigen retrieval and the following steps. Finally, all sections were stained with 4 ‘‐6 ‘‐diamino‐2‐phenylindole (DAPI, cat. D9542, Sigma‐Aldrich) to mark the nuclei. The stained slides were scanned using the Mantra System (PerkinElmer) to visualize, quantify, analyze, and phenotype multiple types of immune cells simultaneously, and the multispectral images were established. Images were analyzed and quantified using inForm tissue analysis software (PerkinElmer). The infiltration z‐score was calculated with R parameter “scale,” and plotted with R package “pheatmap.” Our study reported the density and positive rate of PD‐1, PD‐L1, CD3, and CD8 staining on lymphocytes in parenchymal and stromal compartments. PD‐L1 tumor proportion score (TPS) was calculated, and the cut‐off of positive PD‐L1 expression was 1%.

### 2.3 The landscape of immune cells infiltration in the tumor immune environment

The transcriptome data of RNA‐seq of GDC TCGA Breast Cancer (BRCA) were obtained from the UCSC Xena database (https://gdc‐hub.s3.us‐east‐1.amazonaws.com/download/TCGA‐BRCA.htseq_fpkm.tsv.gz). With TIMER2.0 (http://timer.cistrome.org/),^27^, ^28^, ^29^ tumor immune infiltration estimation of CIBERSORT^30^ for all TCGA‐BRCA tumors was downloaded from TIMER database (http://timer.cistrome.org/infiltration_estimation_for_tcga.csv.gz). Briefly, the CIBERSORT scores of TNBC, HR+, and HER2+ samples were obtained from the above results to obtain a fraction matrix of immune cell infiltration, which estimated the abundances of 22 different leukocytes.

### 2.4 Statistical analyses

Continuous variables were presented as medians with interquartile ranges (IQRs). We performed statistical analysis with R version 4.0.3. Wilcoxon test was used to calculate differences in CIBERSORT scores between groups. The difference in the infiltration of PD‐1, PD‐L1, CD3, and CD8 TILs was analyzed by the Wilcoxon test between two groups. Pearson correlation coefficient was used to assess the associations among infiltrations of different immune cells. The survival curve was estimated by Kaplan–Meier analysis, and a log‐rank test was used to compare survival differences between groups. *p*‐values <0.05 were considered statistically significant.

## 3 RESULTS

### 3.1 Patient characteristics

A total of 81 patients with stage II‐III breast cancers were included in this study, including 27 TNBC, 24 HR+, and 30 HER2+. The median age at diagnosis was 51 years. Patient baseline, tumor, and immune characteristics were summarized in Table 1. Most patients were diagnosed with stage II (54%), and 90% (73 patients) had lymph node metastasis. All patients had no evidence of metastasis at the time of diagnosis. The median Ki‐67 expression was 50%, and positivity ranged from 10% to 80%. All 81 patients were treated with NAT, and the pCR rate after treatment was 26% (21/81). When comparing the clinico‐pathological characteristics of patients with TNBC, HR+ and HER2+ breast cancer, we observed, as expected,^31^, ^32^ a higher proportion of lymph node metastasis at diagnosis in patients with HR+ and HER2+ tumors compared to patients with TNBC (*p* < 0.05). The median Ki‐67 expression of TNBC tumors (60%) was significantly higher than other types.

**TABLE 1:** Patient baseline characteristics

Characteristic	Overall *N* = 81^a^	HR+ *N* = 24^a^	HER2+ *N* = 30^a^	TNBC *N* = 27^a^	*p*‐value^b^
Age	51 (45, 58)	50 (46, 56)	54 (50, 64)	46 (40, 56)	0.027
Length	3.30 (2.60, 4.10)	3.50 (2.60, 5.15)	3.20 (2.73, 3.80)	3.30 (2.30, 4.20)	0.645
Width	2.70 (2.10, 3.60)	2.65 (2.08, 3.88)	2.70 (2.12, 3.10)	2.80 (1.90, 3.50)	0.901
Invasive carcinoma	50 (35, 60)	50 (40, 62)	50 (30, 60)	50 (30, 65)	0.818
TILs	8 (5, 15)	8 (5, 10)	10 (5, 15)	8 (5, 12)	0.569
Ki67	50 (30, 60)	30 (20, 42)	45 (30, 60)	60 (50, 70)	<0.001
Histologic grade					0.198
2	77 (95%)	24 (100%)	29 (97%)	24 (89%)	
3	4 (4.9%)	0 (0%)	1 (3.3%)	3 (11%)	
Vascular tumor thrombus					0.305
0	71 (88%)	19 (79%)	28 (93%)	24 (89%)	
1	10 (12%)	5 (21%)	2 (6.7%)	3 (11%)	
Neural invasion					>0.999
0	80 (99%)	24 (100%)	29 (97%)	27 (100%)	
1	1 (1.2%)	0 (0%)	1 (3.3%)	0 (0%)	
Lymph node metastasis					0.028
0	8 (9.9%)	3 (12%)	0 (0%)	5 (19%)	
1	73 (90%)	21 (88%)	30 (100%)	22 (81%)	
Stage					0.467
II	44 (54%)	11 (46%)	16 (53%)	17 (63%)	
III	37 (46%)	13 (54%)	14 (47%)	10 (37%)	
PDL1 status					0.338
Negative	33 (41%)	9 (38%)	10 (33%)	14 (52%)	
Positive	48 (59%)	15 (62%)	20 (67%)	13 (48%)	
pCR					0.194
N	60 (74%)	21 (88%)	21 (70%)	18 (67%)	
Y	21 (26%)	3 (12%)	9 (30%)	9 (33%)	

^a^ Median (IQR); *n* (%).

^b^ Kruskal–Wallis rank sum test; Fisher's exact test; Pearson's chi‐squared test.

### 3.2 TNBC shows different immune characteristics compared with HR+ and HER2+

**FIGURE 1:**
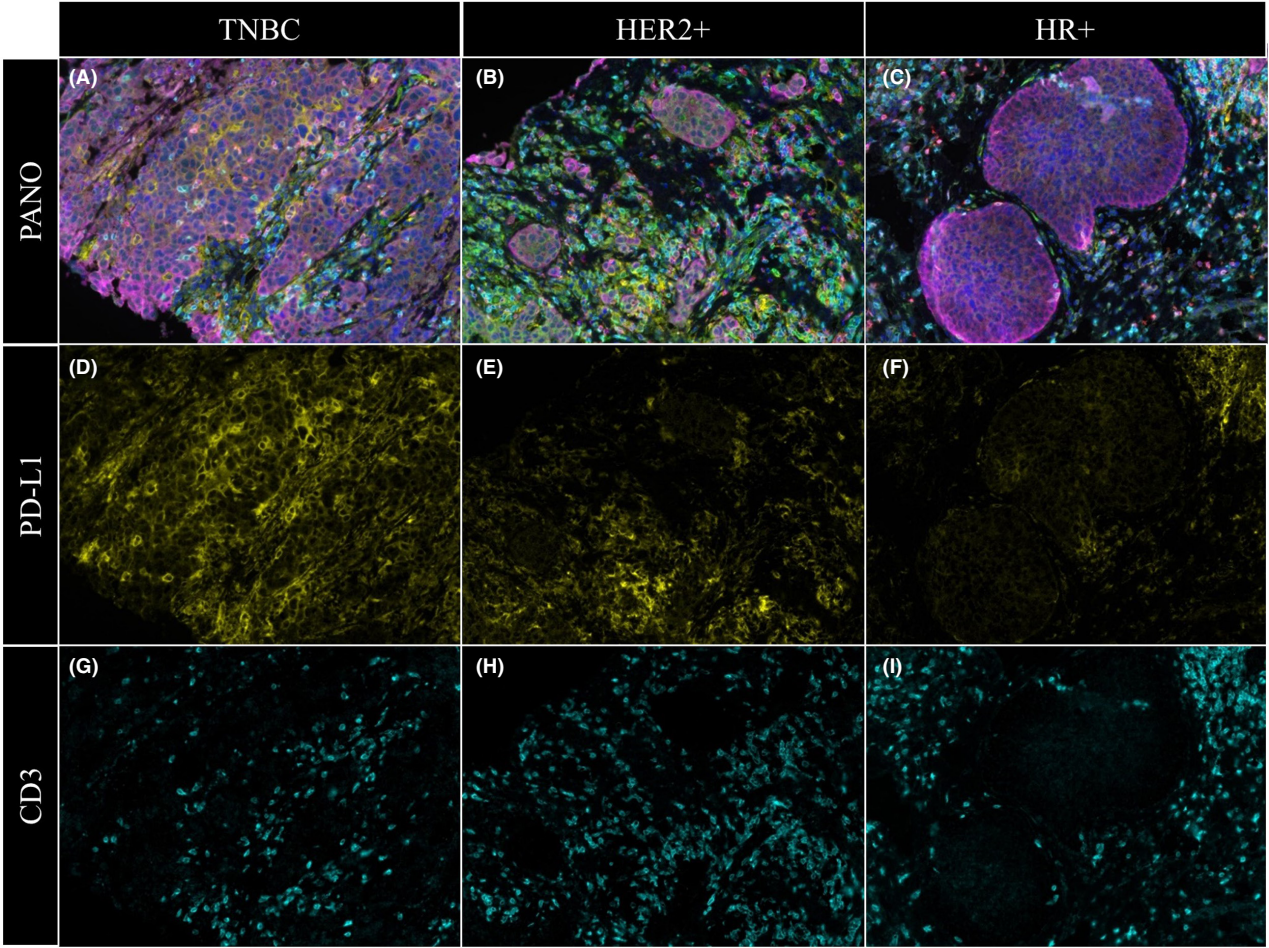
Multiplex staining for PD‐1, PD‐L1, CD3, CD8, and panCK in TNBC, HR+, HER2+ breast cancer tissue. (A–C) Panoramic (PANO) five‐color images showing the expression of PD‐1 (green), PD‐L1 (yellow), CD3 (indigo), CD8 (red), and panCK (purple) DAPI (blue) in TNBC (A), HER2+ (B), and HR+ (C). (D–F) Visualization of PD‐L1 (yellow) under fluorescence. (G–I) Visualization of CD3 (indigo) under fluorescence. Magnification: ×100

**FIGURE 2:**
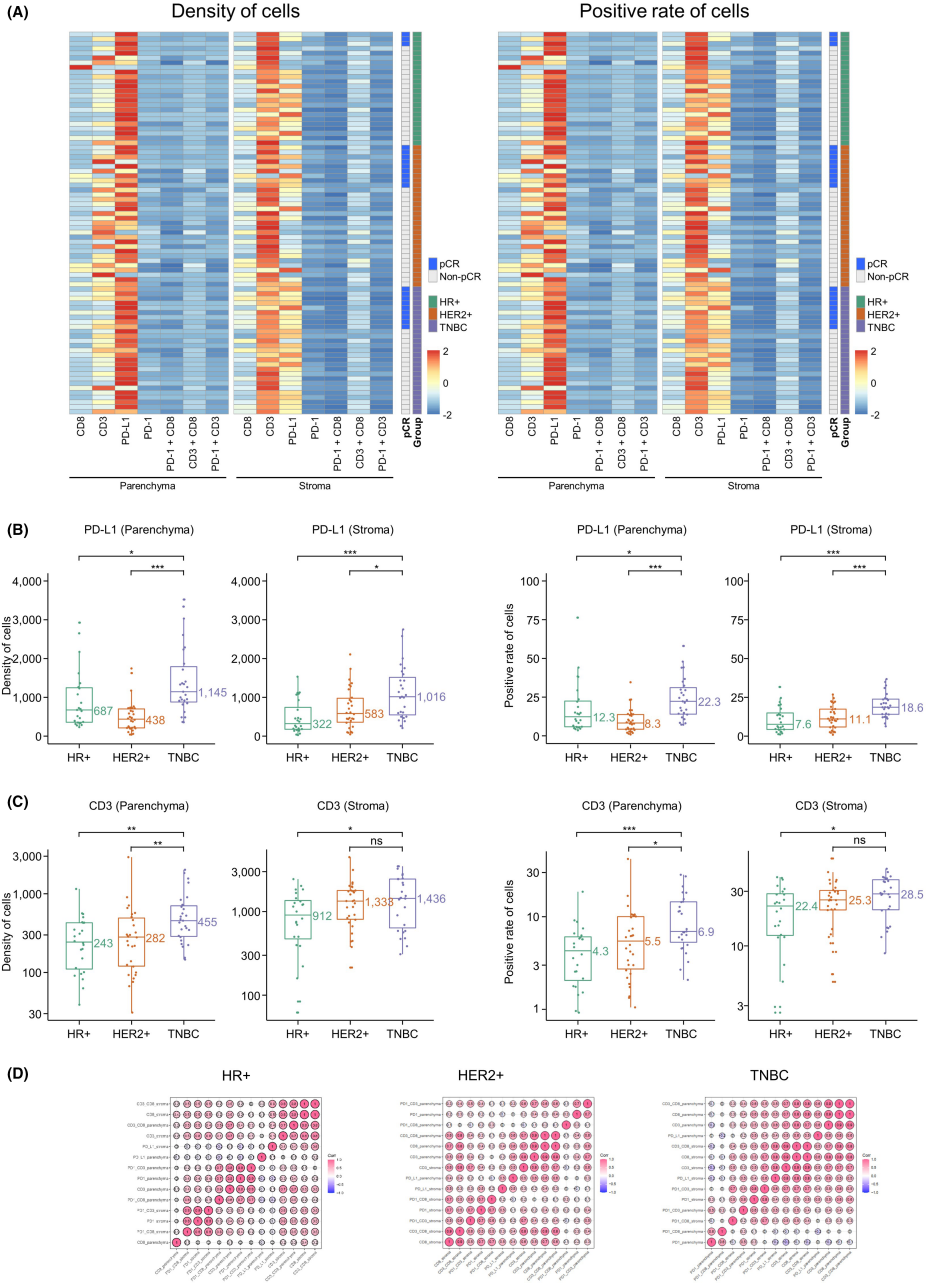
Immune characteristics of three different breast subtypes, HR+, HER2+, and TNBC. (A) The heat map showing the density and positive cell rate of several key immune markers, PD‐1, PD‐L1, CD3, and CD8 detected by immunofluorescence‐based Tissue Microenvironment Analysis Panel (MAP) in 81 BC patients. (B) The PD‐L1 density and positive cell rate difference in three groups in stroma and parenchyma. (C) The CD3 density and positive cell rate difference in three groups in stroma and parenchyma. (D) Correlation analyses of immune markers PD‐1, PD‐L1, CD3, and CD8 in stroma and parenchyma of three different breast subtypes. *, *p* < 0.05; **, *p* < 0.01; ***, *p* < 0.001; ns, no significance

We first evaluated the sTILs and iTILs in pathologic sections among the three groups. TILs infiltration showed no significant differences between three subtypes which were 8%, 10%, and 8%, respectively, in HR+, HER2+, and TNBC tumors (Table 1). We found 48% PD‐L1‐positive patients in the TNBC group and 62% and 67% PD‐L1‐positive patients in HR+ and HER2+ tumors (Table 1). We then compared the density and positive cell rate of four key immune markers, PD‐1, PD‐L1, CD3, and CD8, with multiplex staining of MAP (Figure 1). Pan‐CK was used to distinguish tumor and stroma, which meant pan‐CK‐positive indicated the parenchyma and pan‐CK negative indicated stroma. No significant difference was revealed in the expression of four immune cell markers between TNBC, HR+, and HER2+ parenchyma and stroma (Figure 2A). We nevertheless found significantly higher levels of PD‐L1+ cells in stromal and parenchymal TNBC (*p* < 0.05 for both) based on the density and positive rate of cells (Figures 1D–F and 2B). HR+ and HER2+ patients had significantly lower CD3+ TILs than TNBC (Figures 1G–I and 2C).

Correlation analyses were performed to explore the relationship between immune markers of immune cells. The correlation heat map showed that immune markers in both stromal and parenchymal tumors had a strong positive correlation with each other in TNBC patients but not in HR+ and HER2+ patients (Figure 2D), which implicated better tumor lymphocytes infiltration in TNBC.

### 3.3 PD‐L1 expression is associated with pCR

**FIGURE 3:**
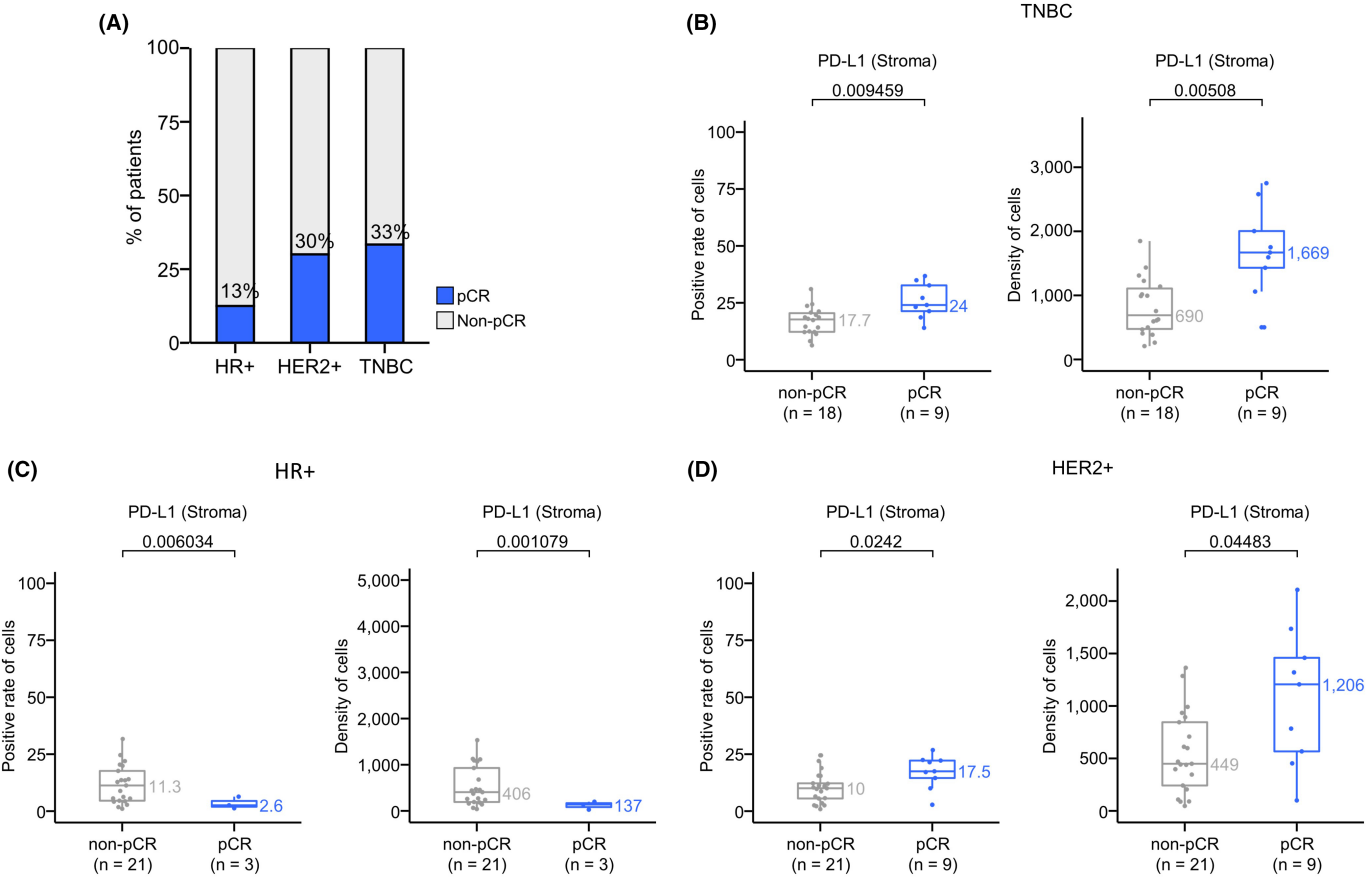
Association of PD‐L1 expression with pCR. (A) pCR ratio following NAT of three groups in 81 patients. (B) Comparision of PD‐L1‐positive cell rate and density in patients who experienced pCR and non‐pCR in stromal and parenchymal TNBC. (C) Comparision of PD‐L1‐positive cell rate and density in patients who experienced pCR and non‐pCR in stromal and parenchymal HR+. (D) Comparision of PD‐L1‐positive cell rate and density in patients who experienced pCR and non‐pCR in stromal and parenchymal HER2+. pCR, pathological complete response

We then explored whether the expression of the selected immune markers was different in pretreatment tumors from patients who experienced a pCR following NAT compared with those from patients who did not. The pCR rate in TNBC patients (33%) was higher than HER2+ (30%) and HR+ (13%) patients (Figure 3A). The pretreatment tumors from patients who experienced pCR after NAT had significantly higher PD‐L1 expression in TNBC based on positive cell rate (*p* = 0.009) and cell density (*p* = 0.005) (Figure 3B). A similar difference was observed in HR+ patients (*p* = 0.006 and 0.001, respectively, Figure 3C) and HER2+ patients (Figure 3D).

### 3.4 The landscape of the tumor immune microenvironment in different subtypes

**FIGURE 4:**
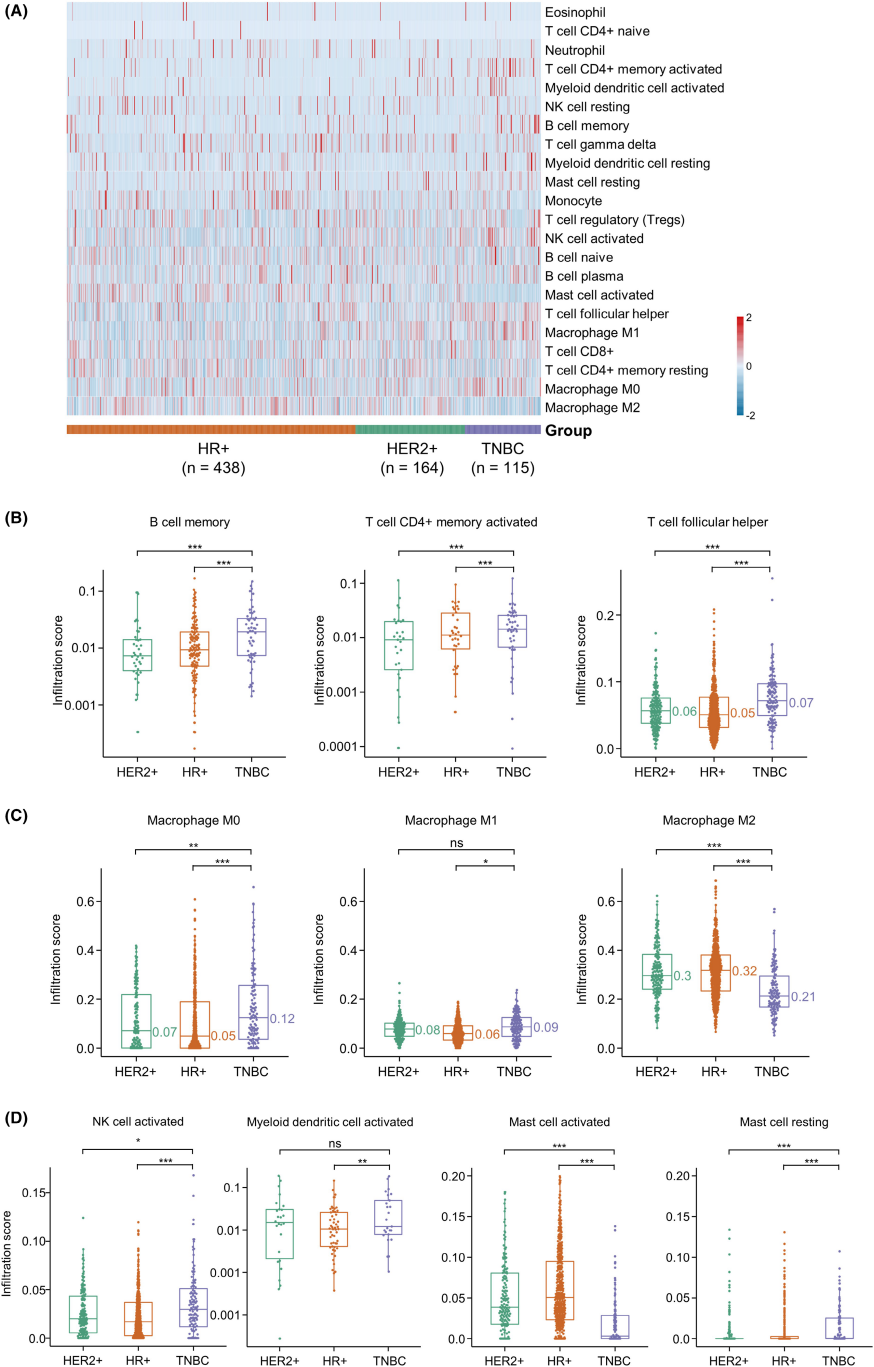
Analysis of 22 immune cell infiltration according to CIBERSORT algorithm in HER2+, HR+, and TNBC. (A) The heat map of tumor immune infiltration estimations of 115 TNBC, 164 HER2+, and 438 HR+ tumors from TCGA breast cancer datasets (BRCA). (B) The infiltration scores of B‐cell memory, T‐cell CD4+ memory activated, and T‐cell follicular helper in HER2+, HR+, and TNBC patients. (C) The infiltration scores of macrophage M0, M1, and M2 in HER2+, HR+, and TNBC patients. (D) The infiltration scores of activated NK cells, activated myeloid dendritic cells, activated mast cells, and resting mast cells in HER2+, HR+, and TNBC patients. *, *p* < 0.05; **, *p* < 0.01; ***, *p* < 0.001; ns, no significance

To further characterize the TME of different breast cancer subtypes, we analyzed the RNA‐seq data of TCGA BRCA, which was available for 115 TNBC, 164 HER2+, and 438 HR+ tumors. We first examined the infiltration score of immune cells in the TME of these breast tumors. The heat map showed corresponding enrichment of the immune signature from the three subtypes (Figure 4A). We found that the Macrophages M2, Macrophages M0, T‐cell CD4 memory resting, and T‐cell CD8+ were among the highest‐proportion immune cells across all the subtypes. While, Eosinophils, T cells CD4 naïve, Neutrophils, and T‐cell CD4+ memory activated are among the lowest‐proportion immune cells. We further revealed the correlation between infiltrating immune cells and different subtypes. The infiltration scores of B cell and T cell, such as B‐cell memory, T‐cell CD4+ memory activated, and T‐cell follicular helper, were higher in TNBC patients than in HER2+ and HR+ patients (Figure 4B). The infiltration score of macrophage M0 was higher, but M2 was lower in TNBC patients, which indicated immunoreactive TME in TNBC (Figure 4C). The infiltration scores of activated NK cells and myeloid dendritic cells whose function enhanced the anti‐tumor immunity were higher in TNBC (Figure 4D). Also, a lower infiltration score of activated mast cells and a higher score of resting mast cells were observed in TNBC (Figure 4D).

### 3.5 High PD‐L1 expression is associated with better survival in TNBC

**FIGURE 5:**
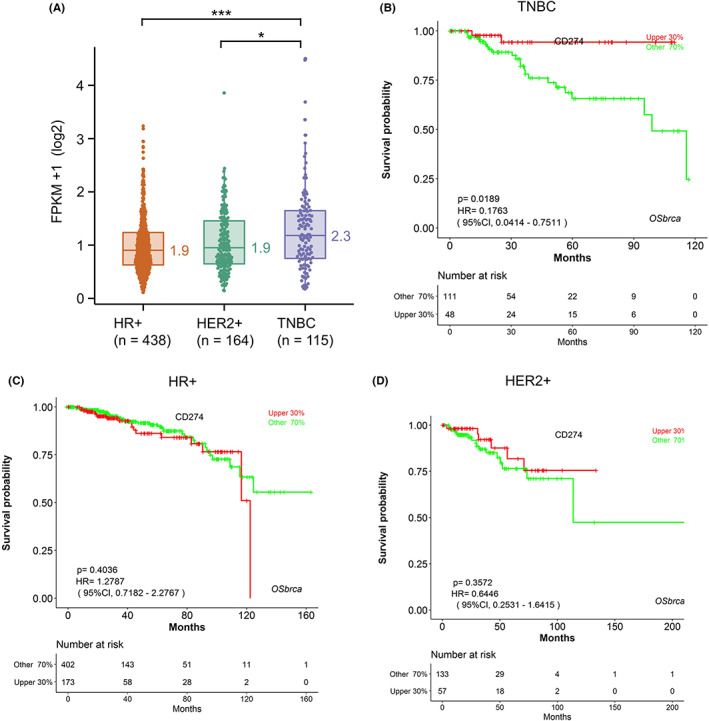
PD‐L1 expression and prognostic value. (A) Expression of PD−L1 (TCGA‐BRCA). Kaplan–Meier curves of the overall survival (OS) based on the PD‐L1 expression in TNBC (B), HR+ (C), and HER2+ (D) patients. *, *p* < 0.05; ***, *p* < 0.001

We further analyzed the average gene expression of immune markers with RNA‐seq data of TCGA BRCA. Consistently with our institutional cohort, we noticed higher PD‐L1 expression in TNBC patients compared to HER2+ and HR+ breast cancers (Figure 5A). To further estimate the prognostic value of PD‐L1, OS was analyzed based on distinct subtypes. Kaplan–Meier plot showed PD‐L1‐positive patients had better survival than PD‐L1‐negative in the TNBC group (*p* = 0.0189, Figure 5B). However, PD‐L1 expression was not associated with the OS in HR+ and HER2+ breast cancers (*p* > 0.05, Figure 5B,C). These findings suggested that the PD‐L1 might serve as a prognostic marker in TNBC.

## 4 DISCUSSION

This study has more fully characterized the TME in different BC subtypes and identified PD‐L1 association with response to NAT. Our study not only examined the expression of PD‐1, PD‐L1, CD3, and CD8 in 81 BC patients who received NAT, but also evaluated how PD‐L1 expression levels correlate with pCR in different subtypes. We found TNBC patients showed significantly different immunoreactive TME compared with HR+ and HER2+ patients. PD‐L1+ cells in tumor and stroma were significantly higher in TNBC patients than in others. PD‐L1+ sTILs were significantly higher in pCR than in non‐pCR patients of all the subtypes who received NAT. High PD‐L1 expression is associated with better survival only in TNBC.

Acknowledging the relatively small sample size, we currently showed that the TME was very different in TNBC compared with HR+ and HER2+ tumors. Our study detected an overall cohort median TIL count of 8% at baseline, with 8% of TNBCs, 8% of HR+, and 10% of HER2+. These results are comparable to several reports in the literature.^15^, ^33^, ^34^ Lymphocyte predominance was not observed in our study, and no case had >50% stromal TILs.

Our MAP analysis showed that PD‐L1+ cell density and rate were significantly higher in both sTILs and iTILs in TNBC patients than in others. Analysis of RNA‐seq data of TCGA BRCA showed similar results. PD‐L1‐positive rate of our institutional TNBC patients was 48%, which is consistent with results for the IMpassion031 trial and IMpassion130 trial, which was 46% and 40.9% respectively.^35^, ^36^ We also observed more CD3+ TILs in TNBC compared to HR+ and HER2+ tumors.

In our study, the cell density and positive cell rate of PD‐L1 TILs before NAT was associated with pCR in all BC patients. Additionally, we analyzed data of TCGA BRCA and found that the PD‐L1‐positive patients had better survival than the PD‐L1‐negative in TNBC patients. PD‐L1 status has significantly affected response to NAT in early‐stage BC patients, especially in TNBC. It has been reported that increased stromal TILs predicted higher rates of pCR to NAT in HER2+ and TNBC.^37^ However, we found no significant increases in baseline TILs cell counts and density of pCR patients.

The difference in the TME for TNBC and other subtypes indicates that TNBC patients could benefit from immunotherapy combined with NAT in TNBC but not in HR+ and HER2+ patients. At present, neoadjuvant chemotherapy is the current standard of care for patients with early TNBC. Three phase III randomized controlled trials are now available to guide the use of immune checkpoint inhibitors combined with neoadjuvant chemotherapy for early‐stage TNBC: KEYNOTE‐522, IMpassion031, and NeoTRIPaPDL1.^38^ KEYNOTE‐522 trial showed neoadjuvant pembrolizumab plus chemotherapy, followed by adjuvant pembrolizumab after surgery, resulted in significantly longer event‐free survival than neoadjuvant chemotherapy alone in stage II‐III TNBC patients.^21^

## 5 CONCLUSION

The TME among different BC subtypes in patients receiving NAT was different, and TNBC patients showed significantly different immunoreactive TME compared with HR+ and HER2+ patients. PD‐L1+ cells in tumor and stroma were significantly higher in TNBC patients than in others. PD‐L1 can be a powerful predictor of pCR after NAT and long‐term survival in TNBC patients. Our characterization of the TME in TNBC suggests a potential role for immunotherapy in these patients.

## AUTHOR CONTRIBUTIONS

Yueping Liu designed the project. Mengxue Han, Yongqiang Yu, and Jinze Li collected clinic samples. Mengxue Han, Si Wu, and Chun Wu analyzed the data. Mengxue Han, Jinze Li, and Yueping Liu wrote the manuscript. All authors approved the final manuscript.

## FUNDING INFORMATION

None.

## CONFLICT OF INTEREST

The authors have no conflict of interest.

## ETHICS STATEMENT

This research was approved by the ethics committee of The Fourth Hospital of Hebei Medical University (no. 2022KY05P).

## Data Availability

The data that support the findings of this study are available from the corresponding author upon reasonable request.
